# Using Dendritic Cell-Based Immunotherapy to Treat HIV: How Can This Strategy be Improved?

**DOI:** 10.3389/fimmu.2018.02993

**Published:** 2018-12-18

**Authors:** Laís Teodoro da Silva, Bruna Tereso Santillo, Alexandre de Almeida, Alberto Jose da Silva Duarte, Telma Miyuki Oshiro

**Affiliations:** Laboratorio de Investigacao em Dermatologia e Imunodeficiencias, Hospital das Clinicas HCFMUSP, Faculdade de Medicina, Universidade de São Paulo, São Paulo, Brazil

**Keywords:** dendritic cells, HIV, immunotherapy, therapeutic vaccine, clinical trial

## Abstract

Harnessing dendritic cells (DC) to treat HIV infection is considered a key strategy to improve anti-HIV treatment and promote the discovery of functional or sterilizing cures. Although this strategy represents a promising approach, the results of currently published trials suggest that opportunities to optimize its performance still exist. In addition to the genetic and clinical characteristics of patients, the efficacy of DC-based immunotherapy depends on the quality of the vaccine product, which is composed of precursor-derived DC and an antigen for pulsing. Here, we focus on some factors that can interfere with vaccine production and should thus be considered to improve DC-based immunotherapy for HIV infection.

## Introduction

Although antiretroviral therapy has deeply improved the quality of life of HIV-infected individuals, some problems must be overcome, such as viral resistance, drug toxicities, therapeutic failure, and lack of drug access to viral reservoirs (1–4), which impair treatment effectiveness and patient adherence and hinder the discovery of functional or sterilizing cures.

In this context, harnessing dendritic cells (DC) to treat HIV infection is a promising strategy that has been extensively studied in recent years (5–21). The rationale for using DC is based on their essential role in the immune system, priming a specific immune response (22, 23). This strategy was initially tested for cancer treatment (24) and then for infectious diseases (5, 25), and more recently, tolerogenic DC have been evaluated for treating autoimmune diseases (26, 27).

Particularly in HIV infection, DC are qualitatively and quantitatively impaired in the host. In fact, HIV is able to evade innate immune sensing by DC, leading to suboptimal maturation that results in a poor antiviral adaptive immune response (28). Thus, the administration of properly sensitized DC can drive the immune response to a specific target, improving the anti-HIV-specific response.

More recent approaches have proposed a strategy using DC to reactivate HIV reservoirs (29, 30) together with a potent antiretroviral drug, which could finally promote the discovery of a long-awaited sterilizing cure. This timely and paramount approach encourages further studies on this type of intervention.

While many studies have demonstrated the high potential of DC-based strategies to stimulate an anti-HIV immune response *in vitro* (31–33), a systematic review of currently published trials concluded that in general, clinical outcomes have been modest, and the expected success rates have not been achieved (34). This outcome suggests that the full potential of this technique has not yet been realized, and opportunities to improve the efficacy of this strategy remain.

In many clinical trials, the patients' vaccine responses are very heterogeneous. Among patients enrolled in the same study and treated with the same strategy, some patients have good clinical outcomes, while others do not present a vaccine response (6, 20), which could be due to the individual characteristics of each patient and/or differences in the final vaccine product.

**Figure 1:**
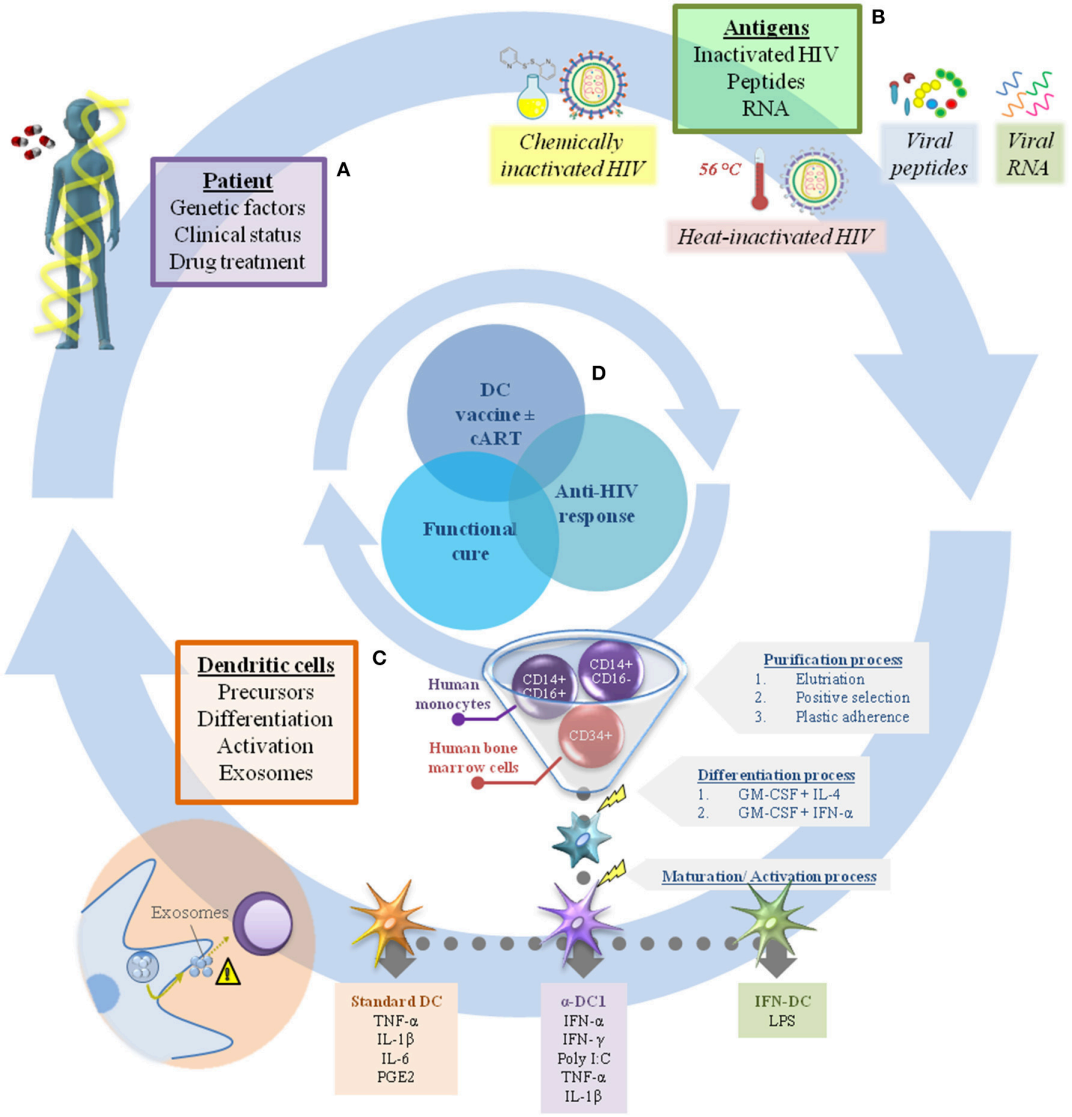
Challenges in dendritic cell immunotherapy for HIV infection. There are many factors that should be considered in the production of DC-based vaccines to achieve a sufficient immune response against HIV combined with viral load control. In general, these can include elements related to the individual patient **(A)**, such as genetic factors, clinical status, and drug treatment (cART interruption or not after receiving the immunization). In addition, the range of antigens available to pulse DC is extensive, making it a challenge to choose the best one **(B)**. The factors related to the vaccine product **(B,C)** are just as important, including the choice of appropriate DC precursors (CD34+ cells or monocytes) and their differentiation/activation protocols (e.g., standard DC, α-DC1, IFN-DC), while also taking into account the potential of DC to produce exosomes (considering their role in regulation of the immune response) **(C)**. In this context, proper assembly of each individual gear could achieve viral infection control and make possible the “functional cure” **(D)**.

In this context, the efficacy of DC-based immunotherapy depends mainly on two factors: (i) the general condition of the patient, which is determined by genetic factors and clinical status; and (ii) the vaccine product, generally composed of monocyte-derived DC (MoDC) and the antigen used to pulse them (Figure 1).

Host genetic and clinical determinants will not be addressed in the present review. Instead, this review will focus on some factors that can interfere with vaccine production and should be taken into account to improve DC-based immunotherapy for HIV infection.

## Clinical Trials

Clinical trials performed thus far have been phase I or phase II trials enrolling from four up to fifty-two patients, who received from one to thirty million DC per dose (5–21). To date, four clinical trials recruited treatment-naïve (5, 6, 11) or untreated (13) HIV-1-infected subjects, while 13 other studies enrolled patients on combined antiretroviral therapy (cART) in which the drug treatment was either interrupted (7, 8, 10, 15–17, 19–21) or not interrupted (9, 12, 14, 18) after patients received the immunization. Analytical treatment interruption is useful to evaluate the effects of DC immunization on viral replication (Table 1).

**Table 1:** Currently published clinical trials on the use of DC-based vaccines for HIV infection.

**Investigator**	**Study phase**	**Number of patients enrolled**	**Treatment**	**Vaccine**							**Vaccine responses**	
				**Dendritic cel**							
				**Protocol for obtaining precursor**	**Type**	**Maturation**	**Quantity per dose**	**HIV antigen**	**Number of doses**	**Route**	**Virological**	**Immunological**
Kundu et al. (5)	I	6	Naive	Isolated monocytes by Percoll gradient	Allogeneic and/or autologous MDDC	None	2 – 8 × 10^6^	rHIV-1 MN gp160 or Gag, Pol and Env	6–9	IV	None	HIV-specific CTL response
Lu et al. (6)	I	18	Naive	Enriched monocytes by plastic adherence	GM-CSF/ IL-4 autologous MDDC	IL-1β, IL-6 and TNF-α	3 × 10^7^	AT-2–inactivated autologous virus	3	SC	Decrease in the HIV RNA load	HIV-specific CTL and CD4+ T cell responses
García et al. (7)	I	18	on cART	Enriched monocytes by plastic adherence	GM-CSF/ IL-4 autologous MDDC	IFN-α	106	Autologous heat-inactivated HIV	4	SC	Decrease in the HIV RNA load	Th1 and HIV-1–specific CD8+ T cell responses
Ide et al. (8)	I	4	on cART	Enriched monocytes by plastic adherence	GM-CSF/ IL-4 autologous MDDC	TNF-α	0,7 – 1,8 × 10^7^	Peptides (Gag, Nef and Env)	6	SC	None	HIV-specific T cell responses
Connolly et al. (9)	I	18	on cART	Enriched monocytes by plastic adherence	GM-CSF/ IL-4 autologous MDDC	IL-1β, IL-6 and TNF-α	1 × 10^6^ to 10 × 10^6^	Peptides (Gag, Pol, Env) and influenza A virus matrix protein peptide	2	IV or SC	Not done	HIV-specific CD8^+^ T cell responses
Gandhi et al. (10)	I	29	on cART	Enriched monocytes by plastic adherence	GM-CSF/ IL-4 autologous MDDC	IL-1β, IL-6, TNF-α and PGE_2_	1,5 – 6 × 10^6^	ALVAC-HIV vCP1452: Viral vector + peptides (Gag, Pol, Nef and Env)	3	SC	None	HIV-specific T cell responses
Kloverpris et al. (11)	I	12	Naive	Enriched monocytes by plastic adherence	GM-CSF/ IL-4 autologous MDDC	IL-1β, IL-6, TNF-α and PGE_2_	1 × 10^7^	Peptides (Gag, Pol, Env, Vpu and Vif)	4	SC	Decrease in the HIV RNA load	HIV-specific CTL and CD4+ T cell responses
Routy et al. (12)	I	10	on cART	Enriched monocytes by plastic adherence	GM-CSF/ IL-4 autologous MDDC	TNF-α, IFN-γ and PGE_2_ plus CD40L	1 × 10^7^	mRNA-transfected (Gag, Vpr, Rev and Env)	4	ID	None	CD8^+^ T cell proliferative response to HIV antigens
García et al. (13)	I	24	Untreated for at least the 2 years before enrollment	Enriched monocytes by plastic adherence	GM-CSF/ IL-4 autologous MDDC	IL-1β, IL-6 and TNF-α	≤ 8 × 10^6^	Autologous heat-inactivated HIV	3	SC	Decrease in the HIV RNA load	HIV-specific T cell responses
Van Gulck et al. (14)	I/II	6	on cART	Isolated monocytes by immunomagnetic selection of CD14+	GM-CSF/ IL-4 autologous MDDC	TNF-α and PGE_2_	1 × 10^7^	mRNA-transfected (Gag or Tat-Rev-Nef)	4	ID and SC	inhibition of HIV-1 IIIB replication *in vitro*	T cell proliferation and HIV-specific T cell responses
Allard et al. (15)	I/IIa	17	on cART	Enriched monocytes by plastic adherence	GM-CSF/ IL-4 autologous MDDC	IL-1β, IL-6, TNF-α and PGE_2_	1 × 10^7^	mRNA-transfected (Tat, Rev or Nef)	4	ID and SC	None	HIV-specific CTL and CD4+ T cell responses
García et al. (16)	I/II	36	on cART	Enriched monocytes by plastic adherence	GM-CSF/ IL-4 autologous MDDC	IL-1β, IL-6, TNF-α and PGE_2_	1 × 10^7^	Autologous heat-inactivated HIV	3	ID or SC	Decrease in the HIV RNA load	CD4^+^ CD38^+^ HLADR^+^ T cells and T cell IFN-γ production
Levy et al. (17)	I	19	on cART	Enriched monocytes by elutriation	GM-CSF/IFN-α autologous MDDC	LPS	1,5 × 10^7^	HIV LIPO5 peptides (Gag, Pol and Nef)	4	SC	Decrease in the HIV RNA load	Polyfunctional HIV specific T cells
Gandhi et al. (18)	I/II	15	on cART	Enriched monocytes by plastic adherence	GM-CSF/ IL-4 autologous MDDC	IL-1β, IL-6, TNF-α and PGE_2_	1.5–6 × 10^6^	mRNA-transfected (Gag and Nef) or/and KLH pulsed	4	ID	Not done	T cell proliferation
Jacobson et al. (19)	IIB	52	on cART	Enriched monocytes by plastic adherence	GM-CSF/ IL-4 autologous MDDC	TNF-α, IFN-γ, PGE_2_ and CD40L	~1 × 10^7^	mRNA-transfected (Gag, Vpr, Rev and Nef)	4	ID	None	CD8+CD28+/CD45RA-effector memory CTL
Macatangay et al. (20)	I/II	11	on cART	Enriched monocytes by elutriation	GM-CSF/ IL-4 autologous	TNF-α, IL-1β, IFN-α, IFN-γ, and poly I:C	1 × 10^7^	Autologous, inactivated, HIV-1–infected apoptotic cells	4	SC	Decrease in the HIV RNA load	T-cell activation
Gay et al. (21)	I	6	on cART	Enriched monocytes by plastic adherence	GM-CSF/ IL-4 autologous MDDC	TNF-α, IFN-γ, PGE_2_ and CD40L	1.2 × 10^7^	mRNA-transfected (Gag, Vpr, Rev and Nef)	5	ID	None	CD28+/CD45RA- CD8+ memory and CD28-/CCR7-/CD45RA- CD8+ effector T cell responses

*cART, combine antiretroviral therapy; IV, intravenous; SC, subcutaneous; ID, intradermal*.

Overall, immunotherapy trials present high variability in terms of the protocol used to obtain DCs, the number of doses, patient profiles and the immunization route. In this regard, the only commonality between currently published DC-based HIV vaccines is that the DC used in all protocols have been derived from monocytes because they are easy to obtain (Figure 1). However, despite the variability in study design, DC immunotherapy has been shown to be well-tolerated and safe, with only minor and transient side effects, including fever (8, 9, 14), enlargement of local lymph nodes (8, 13, 16), mild local redness (14–16) and flu-like symptoms (7, 13, 16).

Clinical outcomes were also highly variable between studies. In some, decreased plasma viral loads were observed in HIV-infected vaccinated individuals, but specific immune responses were usually transitory (6, 7, 11, 13, 16, 17). When the effects of immunotherapy on activation markers were monitored, CD38, and human leukocyte antigen (HLA)–DR expression increased on T cells (16, 20). In addition, eight out of seventeen trials showed that some individuals exhibited HIV-specific T cell responses that were not associated with decreased viral load or virologic control (5, 8, 10, 12, 15, 19–21).

These different outcomes may have been influenced by the protocols used to generate the vaccine products and their quality (MoDC maturation cocktail, HIV antigen used to pulse MoDC, quantities of cells inoculated per dose and number of doses administered) as well as patients' individual characteristics (e.g., CD4^+^ T cell nadir (35), HLA alleles (36), and polymorphisms in genes involved in immune modulation (37–41).

The combination of factors discussed above may have affected the immune responses of vaccinated patients, which could explain why some of these individuals did not respond to immunotherapy (40).

## Challenges in MoDC Preparation

### Cell Precursors

Myeloid DC can be detected at a reduced frequency in peripheral blood. For immunotherapeutic protocols in which large numbers of cells are required, DC may alternatively be differentiated from precursors, such as CD34^+^ hematopoietic progenitor cells and CD14^+^ monocytes present in peripheral blood (42, 43).

Considering that the number of peripheral blood DC is low and that differentiation techniques require complex generation methods, only a small number of clinical trials, all related to cancer, have been published using DC generated from CD34^+^ cells (44, 45); thus, MoDC are the most commonly used cells in a wide range of clinical applications (5–21, 46).

Monocytes are highly plastic cells that can alter their phenotype according to signals present in the microenvironment; for example, they may differentiate into MoDC under inflammatory conditions (43). MoDC have a high capacity for antigen presentation and naive T lymphocyte stimulation, similar to DC generated from CD34^+^ cells (47).

Three circulating monocyte subsets have been described in human blood: classical (CD14^++^CD16^−^), intermediate (CD14^++^CD16^+^), and non-classical (CD14^+^CD16^++^) (48). Increased numbers of inflammatory CD16^+^ monocytes are found in HIV-infected individuals (49, 50) and can act as targets for HIV entry through the highly expressed CCR5 (an HIV co-receptor); these monocytes may be more permissive to productive HIV infection than other monocyte subtypes (51).

While MoDC generated from CD16^+^ monocytes secrete increased amounts of TGF-β1, MoDC generated from CD16^−^ monocytes produce more of the IL-12p70 cytokine (52). In this context, considering that all three monocyte subtypes can be differentiated into MoDC *in vitro* and that the MoDC generated have distinct phenotypic and functional abilities (53), selection of the monocytic precursor may substantially influence vaccine performance. In fact, it was shown that “CD16^+^” MoDC-stimulated T cells produce more IL-4 than lymphocytes co-cultured with MoDC obtained from CD16^−^ monocytes; therefore, “CD16^+^” MoDC can polarize the naive T cell response toward the Th2 phenotype (53). In the context of anti-HIV therapy, obtaining MoDC that secrete IL-12p70 is desirable for inducing IFN-γ-producing T lymphocytes (Th1 profile) (54). In the future, developing a clinical-scale procedure to enrich the non-classical monocyte subset could be a promising option.

Another important point to consider is the technique used to acquire peripheral blood cells. To obtain a large number of monocytes, leukapheresis is first performed, followed by an additional step to isolate or enrich the monocyte population [elutriation (55) or positive purification by CD14^+^ microbeads or adherence to plastic (56)].

During leukapheresis, a higher centrifuge speed yields residual platelets (57), which may subsequently attach to the monocytes and induce the production of cytokines (IL-1α and TNF-α) (58), pre-activating monocytes that could impair their differentiation into DC after *in vitro* stimulation. In addition, if leukapheresis itself leads to platelet activation, HIV-infected patients, even those receiving cART, may exhibit basal activation of these blood cells (59–61), which interfere with monocyte functionality and induce DC activation *in vitro*, affecting DC-mediated T lymphocyte polarization (62, 63).

Furthermore, if the monocytes are obtained by plastic adherence, the adhesion capacity of the platelets can reduce their yield, which subsequently reduces the yield of MoDC.

Considering the factors discussed above, vaccine product quality can be directly influenced by the first stages of DC acquisition, such as the leukapheresis process and the monocyte subsets used.

### MoDC Differentiation/Activation Protocols

MoDC-based immunotherapy requires custom conditions for producing mature MoDC capable of stimulating an appropriate immune response. For this reason, protocols should be guided by factors that contribute to viability, migration, co-stimulatory molecule expression, cytokine secretion, antigen presentation and T cell stimulation (64).

Although IL-4 and GM-CSF are used for MoDC differentiation in most studies, different concentrations or cytokine arrangements have been used in clinical trials (16, 17, 20), resulting in different vaccine products with variable performance once these cells present high plasticity.

Another important point to consider is maturation/activation stimuli. Correct insight is fundamental because the product has the potential to “educate” MoDC behavior. The commonly used maturation cocktail for MoDC comprises the proinflammatory cytokines TNF-α, IL-1ß, and IL-6 combined with PGE2, which was established as the “gold standard” for MoDC maturation (the so-called “standard DC” or “sDC”) (65). sDC upregulate major histocompatibility complex (MHC) class I and II molecules, co-stimulatory molecules and CCR7 but fail to induce IL-12p70 production, probably due to PGE2 (66–68). The removal of PGE2 from these cocktails generates MoDC with similar profiles but low CCR7 expression and subsequent decreased migration to the lymphoid organs (69). The combination of different cytokines induces distinct responses, reflecting the complexity involved in establishing an effective protocol. In fact, sDC (with or without PGE2) have been adopted in most MoDC-based HIV immunotherapy protocols (Figure 1).

To improve the performance of sDC, alternative strategies have been developed. For example, type I and II interferons have been used to supplement standard activation stimuli to obtain polarized DC, called alpha-type-I polarized DC (α-DC1), driving a potent Th1 response (54). Recently, α-DC1 were used in a clinical trial for the treatment of HIV-infected individuals after stimulation with autologous HIV-infected apoptotic cells (ApB-DC vaccine). Although safe and immunogenic, only a modest decrease in the HIV-1 RNA load set point was observed after vaccination, and this was not sustained after cART discontinuation. Suboptimal DC function, evidenced by low IL-12 production, was attributed to this modest outcome (20).

Recently, a cancer research group developed “self-differentiated myeloid-derived antigen-presenting cells reactive against tumors DC” (Smart-DC), which are generated via the genetic reprogramming of monocytes. For production, monocytes are transduced with lentiviral vectors co-expressing GM-CSF and IL-4 and a melanoma self-antigen, allowing their self-differentiation into DC, which express typical DC surface molecules and stimulate antigen-specific CTL responses (70). This interesting and innovative approach could be a potential option for future antiviral immunotherapy applications.

Another promising strategy is a preclinical evaluation of an mRNA-electroporated MoDC-based therapeutic vaccine against HIV-1-encoding activation signals (TriMix: CD40L + CD70 + caTLR4 -activated form of TLR4) combined with rationally selected antigen sequences of Gag, Pol, Vif and Nef (HTI—HIV T cell immunogen). *In vitro* assays demonstrated MoDC with appropriate maturation profiles and the ability to induce T cell responses, especially CD8^+^ T cells (71).

Overall, these studies aim to reach the same goal of developing a protocol that can induce the best MoDC capable of eliciting a sufficiently potent immune response that is reproducible *in vivo* and also controls viral replication. Several combinations of differentiation and activation factors are available, but the search for an ideal MoDC continues, and a gold standard protocol for generating successful MoDC-based therapeutic vaccines for HIV-infected individuals has not been established.

### Exosomes

Exosomes have emerged as potential modulators of the immune response in a DC-based immunotherapy context. As a type of extracellular vesicle, exosomes are endocytic-originating small particles (30-100 nm in diameter) composed of lipids that are released by cells into the extracellular environment by the fusion of internal multivesicular compartments. Exosomes participate in intercellular communication via the transfer of a variety of molecules, such as lipids, proteins, DNA, mRNA, and microRNA (72–74).

Many cells, such as neurons, tumors and immune cells, are capable of releasing exosomes. In particular, DC-derived exosomes can express class I and II MHCs, adhesion and co-stimulatory molecules, enabling their ability to directly activate CD8 and CD4 T cells (75–77).

Interestingly, in the context of HIV infection, the exosome dissemination pathway converges to capture and transfer HIV particles via mature DC, suggesting that HIV exploits this pathway to mediate T lymphocyte transinfection (78). Additionally, exosomes derived from HIV-infected DC can transmit HIV to T cells (79) and are capable of inducing the activation of resting primary CD4^+^ T lymphocytes as well as reactivating the HIV-1 reservoir (80). These findings illustrate the close relationship between exosomes and HIV in the DC therapy context.

Considering that whole HIV particles are used in some DC-based immunotherapy protocols, the role of exosomes in DC performance should be considered. After pulsing DC with HIV, DC-derived exosomes were shown to induce the apoptosis of neighboring CD4 T lymphocytes, which has the potential to impair specific anti-HIV immune responses (81). In line with this, preliminary data suggested that exosomes may play a role in modulating the immune response during anti-HIV DC-based immunotherapy. Using gene expression analysis of an exosome marker, it was hypothesized that low exosomal release is more beneficial for DC-based immunotherapy responses than high exosomal release (82), which is an important point that should be considered when using whole viral particles to pulse DC.

Although systematic analyses have not considered the role of exosomes in anti-HIV DC-based clinical trial outcomes, studies have demonstrated a relevant function for these vesicles in immune response regulation. In the context of cancer research, if on one hand tumor-derived exosomes can directly activate specific immune responses and improve anti-cancer responses (83), on the other hand these exosomes can create an immunosuppressive pro-tumorigenic microenvironment, which allows the disease to progress (84). Similarly, in an anti-HIV context, this duality may also be present and should be considered in future trials.

## Antigens and Challenges in Dc-Loading Strategies

A fundamental aspect of DC-based immunotherapy is the selection of the antigen to be incorporated, a decision that must consider the safety and efficacy originating from the effects of the antigen's interaction with DC during the pathogenesis of infection in addition to knowledge of HIV structure.

Although whole particles (inactivated or attenuated) advantageously have greater epitope diversity, they have pathogenic potential due to the virus-cell interaction.

Attenuated viral particles represent one of the most potent known immunogens (85). Research on and development of an anti-HIV vaccine has previously incorporated attenuation procedures, but this approach was abandoned for safety reasons (86). With modern technologies, research examining this process has resumed, and such a strategy potentially represents another DC-based immunotherapy option (87, 88).

The deleterious effects arising from the interaction of viral particles with DC can be minimized by using killed whole HIV particles as an antigen. The consequences of such an approach will depend on the methodology used for chemical or heat inactivation (89–91), which may or may not alter the virus structure and will also influence the type of immune response induced (92).

When using HIV fragments as antigens, the risk of deleterious effects on the individual is lower (although persistent) relative to that posed by using the entire viral particle. However, an even more significant challenge associated with using HIV fragments is the selection of which one to use. The lack of immune protection correlates in HIV infection imposes an unprecedented degree of difficulty on this definition when determining the composition of the product for intervention (93, 94).

Groups have studied the immunogenic potential of DC transfected with mRNA encoding HIV proteins *in vitro* (71, 95, 96) and *in vivo* (12, 14, 15, 18, 19, 21). Some advantages of using RNA as an antigen include the absence of a biological risk of infection and the possibility of designing a sequence restricted to targeting MHC class I or II molecules, thus activating immune responses to CD8^+^ T cells or both CD4^+^ and CD8^+^ T cells, respectively.

Another strategy is the use of the HIV fragments Gag, Tat, Rev, Nef and Vpr, which are commonly employed because they are more conserved than other proteins and are known to induce a T cell immune response (97). However, Nef interferes with DC functionality (98); for this reason, reduced quantities of Nef RNA are used in immunotherapeutic protocols. Similarly, the Vpr gene is truncated to remove its ability to impair DC expression of co-stimulatory molecules and production of the cytokine IL-12 (12, 99).

In clinical trials, both consensus viral sequences for a cohort of patients (14, 15, 18) or mRNA constructs personalized for each individual (12, 19, 21) have been used as immunogens. Although researchers have observed the induction of HIV-specific T cell responses *in vivo* as increases in T cell proliferation (12, 14) and enhancements in effector/memory CD8^+^ T cell responses (15, 19, 21), there has been no sustained impact on patient viral load.

Another strategy is loading DC with DNA encoding HIV antigens. The use of DNA constructs as antigen vectors may overcome difficulties associated with MHC haplotype and peptide mismatches. Additionally, DNA can express antigens with natural posttranslational modifications (100). Moreover, DNA vaccines present several advantages, such as safety, potential to elicit both humoral and cellular immune responses and low cost (101). In this sense, plasmid DNA can be efficiently combined with DC to induce a specific immune response, as demonstrated *in vitro* (33, 102), representing a promising strategy to improve DC-based vaccines.

## Summary

Overall, this review highlights some emerging factors that should be considered to improve the production of vaccines for anti-HIV DC-based immunotherapy protocols. Approaches to improve anti-HIV immunotherapy are complex and challenging. HIV infection is characterized by a chronic immune activation state, a consequence of intense immune stimulation and sustained inflammation, which promotes massive immune cell loss (103, 104). Given that immunotherapy aims to induce the patient's immune response, treatment requires an equilibrium between stimulating a specific immune response to fight the virus and limiting the immune activation state to avoid “adding fuel to the fire.” Additionally, depending on the patient's clinical status, DC precursors as well as effector cells may be committed, which could interfere with the performance of vaccine products.

## Author Contributions

TO conceived the study. AdA, BS, LdS and TO discussed, wrote, and edited the manuscript, and BS also contributed to figure construction. AD provided intellectual guidance. All authors have read and approved the final manuscript.

### Conflict of Interest Statement

The authors declare that the research was conducted in the absence of any commercial or financial relationships that could be construed as a potential conflict of interest.
